# Supplementary material for: The Prevalence of Hypogonadism and the Effectiveness of Androgen Administration on Body Composition in HIV-Infected Men: A Meta-Analysis
Source: Cells. 2021 Aug 12;10(8):2067. doi: 10.3390/cells10082067 (PMC8392149; doi:10.3390/cells10082067)
Supplement: Supplementary file 1 [file cells-10-02067-s001.zip › cells-1342077-supplementary.pdf]

Study	Selection bias	Study design	Data collection	Global rating
**Dobs et al, 1988**	Weak	Observational Longitudinal	Moderate	Moderate
**Croxson et al, 1989**	Strong	Observational Case-control	Moderate	Low
**Raffi et al, 1991**	Weak	Observational Cross-sectional	Moderate	Moderate
**Laudat et al, 1995**	Strong	Observational Cross-sectional	Moderate	Moderate
**Dobs et al, 1996**	Weak	Observational Case-control	Moderate	Moderate
**Grinspoon et al, 1996**	Strong	Observational Cross-sectional	Strong	Moderate
**Schürmeyer et al, 1997**	Weak	Observational Longitudinal	Moderate	Moderate
**Bhasin et al, 1998**	Weak	RCT	Strong	High
**Arver et al, 1999**	Moderate	Observational Case-control	Strong	Moderate
**Dobs et al, 1999**	Weak	RCT	Strong	High
**Kopicko et al, 1999**	Weak	Observational Cross-sectional	Moderate	Moderate
**Salehian et al, 1999**	Strong	Observational Longitudinal	Moderate	Low
**Rietschel et al, 2000**	Strong	Observational Cross-sectional	Moderate	Low
**Rabkin et al, 2000**	Weak	RCT	Moderate	Moderate
**Grinspoon et al, 2000**	Weak	RCT	Moderate	Moderate
**Hadigan et al, 2000**	Weak	RCT	Strong	High
**Biglia et al, 2004**	Strong	Observational Case-control	Moderate	Low
**Andersen et al, 2007**	Strong	Observational Cross-sectional	Moderate	Low
**Crum-Cianflone et al, 2007**	Weak	Observational Cross-sectional	Moderate	Moderate
**Mulligan et al, 2007**	Weak	RCT	Strong	High
**Knapp et al, 2008**	Weak	RCT	Moderate	Moderate
**Teichmann et al, 2008**	Moderate	Observational Cross-sectional	Moderate	Moderate
**Moreno-Pérez et al, 2010**	Weak	Observational Cross-sectional	Moderate	Moderate
**Meena et al, 2010**	Weak	Observational Cross-sectional	Moderate	Moderate
**Rochira et al, 2011**	Weak	Observational Cross-sectional	Strong	Moderate
**Pepe et al, 2012**	Weak	Observational Cross-sectional	Strong	Moderate

**Sunchatawirul et al, 2012**	Weak	Observational Cross-sectional	Moderate	Moderate
**Zona et al, 2012**	Weak	Observational Case-control	Moderate	Moderate
**Guaraldi et al, 2012**	Weak	Observational Cross-sectional	Strong	Moderate
**De Ryck et al, 2013**	Weak	Observational Cross-sectional	Moderate	Moderate
**Pérez et al, 2013**	Weak	Observational Cross-sectional	Moderate	Moderate
**Pepe et al, 2014**	Weak	Observational Cross-sectional	Moderate	Moderate
**Bhatia et al, 2015**	Weak	Observational Cross-sectional	Weak	Low
**Pathak et al, 2015**	Weak	Observational Longitudinal	Moderate	Moderate
**Rochira et al, 2015**	Weak	Observational Cross-sectional	Strong	Moderate
**Gomes et al, 2016**	Weak	Observational Cross-sectional	Weak	Low
**Santi et al, 2016**	Weak	Observational Cross-sectional	Strong	Moderate
**Bajaj et al, 2017**	Weak	Observational Case-control	Weak	Low
**Dutta et al, 2017**	Weak	Observational Cross-sectional	Moderate	Moderate
**Price et al, 2019**	Weak	Observational Cross-sectional	Strong	Moderate
**Bajaj et al, 2020**	Weak	Observational Case-control	Moderate	Moderate
**Pezzaioli et al, 2020**	Weak	Observational Cross-sectional	Moderate	Moderate
**de Vincentis et al, 2021**	Weak	Observational Cross-sectional	Strong	Moderate
**Postel et al, 2021**	Moderate	Observational Cross-sectional	Weak	Low
**Quiros-Roldan et al, 2021**	Weak	Observational Cross-sectional	Moderate	Moderate
**Pilatz et al, 2021**	Weak	Observational Longitudinal	Moderate	Moderate

Supplementary Table S1. Quality assessment of the clinical studies included in the meta-analysis

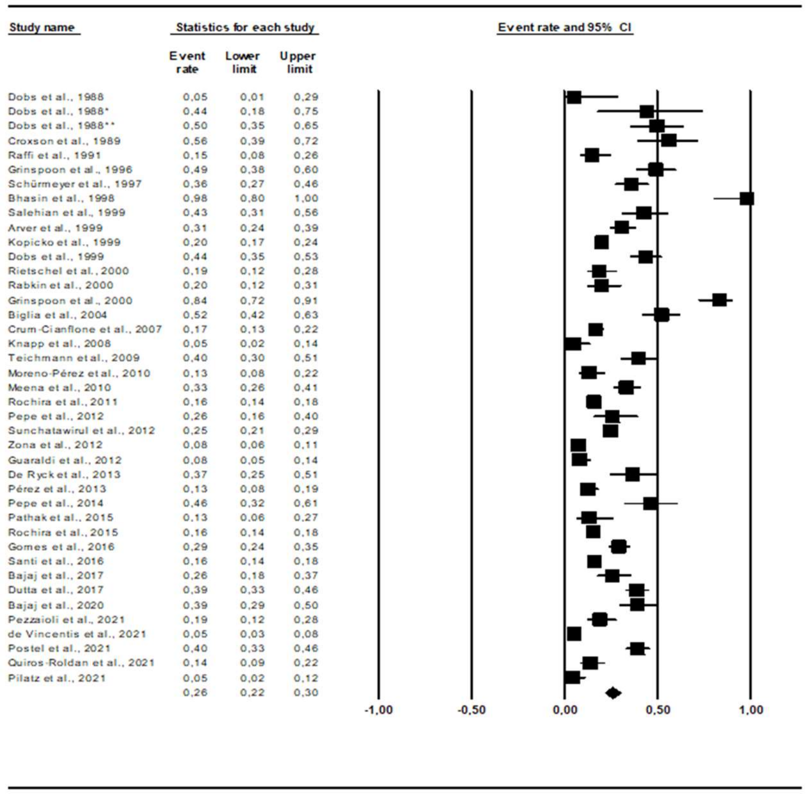

A

Panel A

B

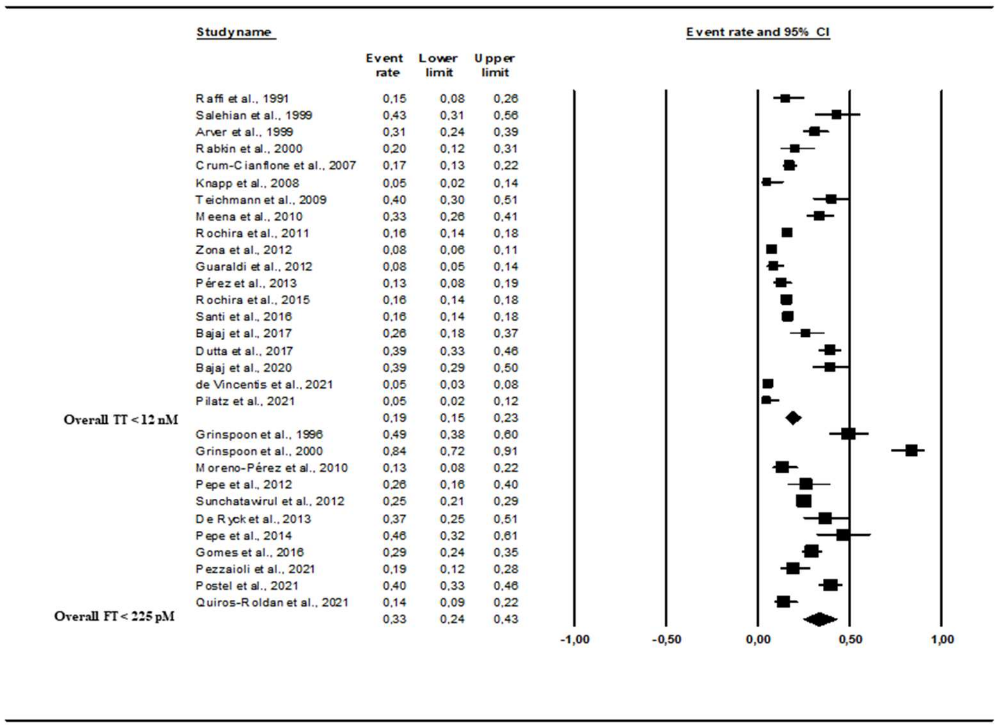

Panel B

Supplementary Figure S1.(Panel A Panel B)

A

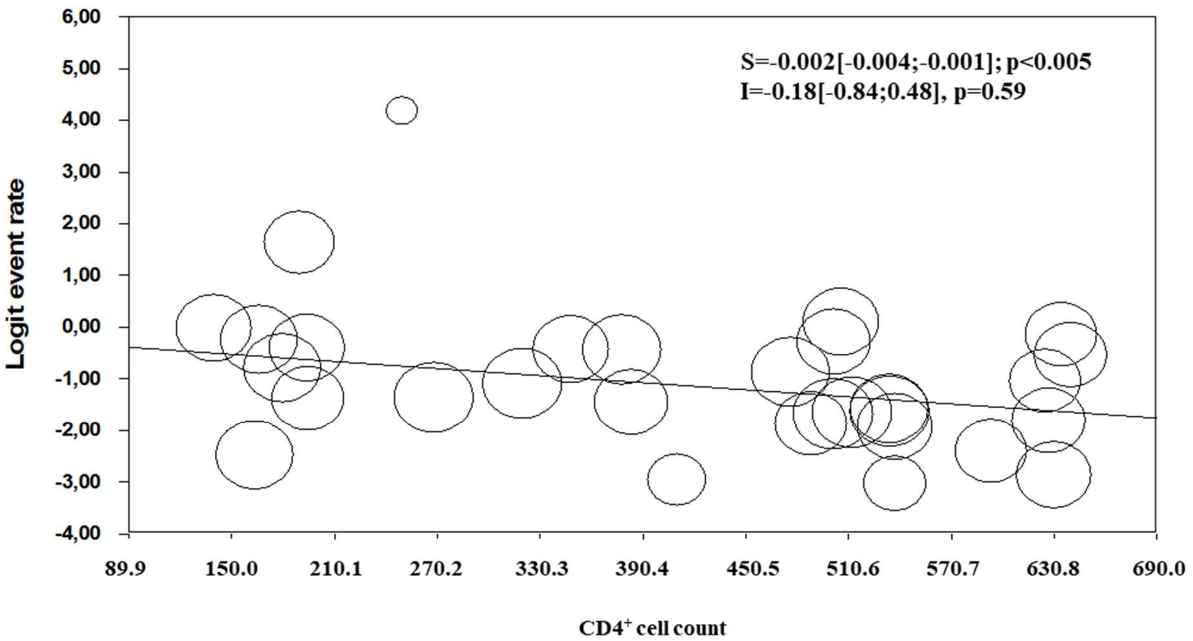

Panel A

B

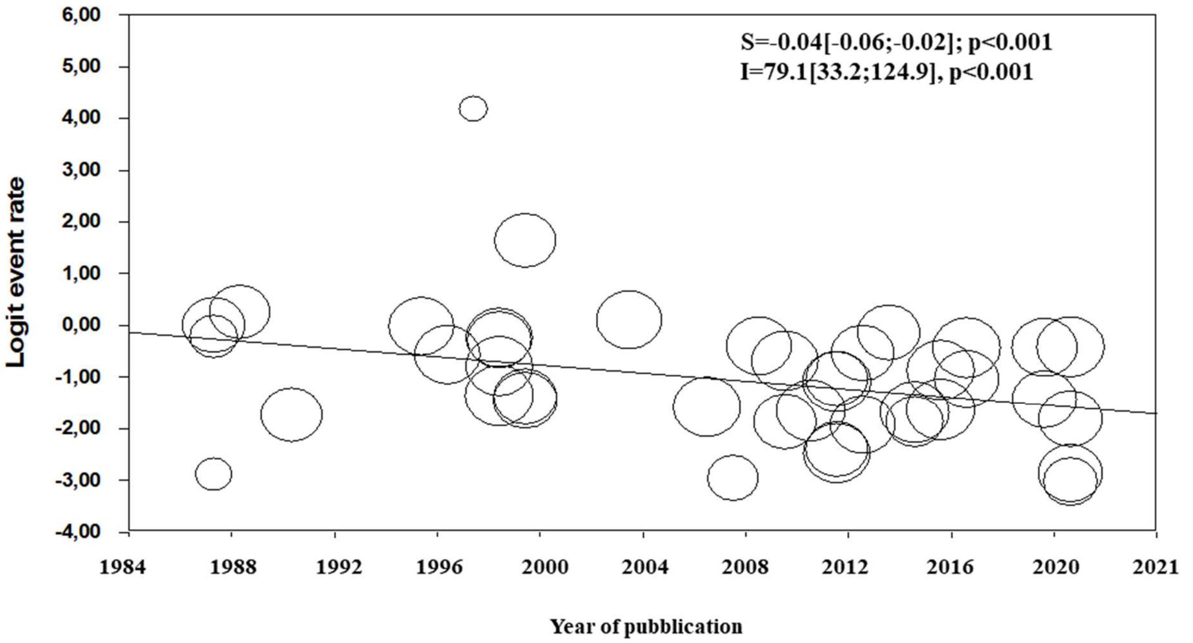

Panel B

Supplementary Figure S2.(Panel A Panel B)

Study or Subgroup	Study group Mean	SD	Total	Control group Mean	SD	Total	Weight	Mean Difference IV, Random, 95% CI	Year
**1.1.1 Androgen-related molecules**									
Strawford 1999	-9.85	1.21	7	2.29	1.91	7	9.6%	-12.14 [-13.81, -10.47]	1999
Storer 2005	-10.7	0.3	43	1.7	0.9	22	9.7%	-12.40 [-12.79, -12.01]	2005
Poretsky 2006	0	0.34	31	0.1	0.34	38	9.7%	-0.10 [-0.26, 0.06]	2006
**Subtotal (95% CI)**			**81**			**67**	**29.0%**	**-8.20 [-18.00, 1.60]**	
Heterogeneity: Tau² = 74.78; Chi² = 3453.59, df = 2 (P < 0.00001); I² = 100%									
Test for overall effect: Z = 1.64 (P = 0.10)									
**1.1.2 Testosterone**									
Bhasin 1998	3.78	0.52	14	0.38	2.19	18	9.7%	3.40 [2.35, 4.45]	1998
Grinspoon 1998	17.5	17	26	2	6.1	26	8.7%	15.50 [8.56, 22.44]	1998
Dobs 1999	9.61	1.49	67	1.04	8.05	66	9.6%	8.57 [6.60, 10.54]	1999
Grinspoon 2000	14.7	12	24	1.6	7.1	26	9.0%	13.10 [7.58, 18.62]	2000
Bhasin 2000	4.5	3.1	15	-1.2	2.08	12	9.6%	5.70 [3.74, 7.66]	2000
Fairfield 2001a	14.7	1	24	1.5	0.1	26	9.7%	13.20 [12.80, 13.60]	2001
Mulligan 2007	-6.1	9.82	41	-12.3	61.15	38	5.0%	6.20 [-13.47, 25.87]	2007
Knapp 2008	24.98	2.98	30	0.51	0.72	31	9.7%	24.47 [23.37, 25.57]	2008
**Subtotal (95% CI)**			**241**			**243**	**71.0%**	**11.61 [6.09, 17.12]**	
Heterogeneity: Tau² = 55.41; Chi² = 817.24, df = 7 (P < 0.00001); I² = 99%									
Test for overall effect: Z = 4.12 (P < 0.0001)									
**Total (95% CI)**			**322**			**310**	**100.0%**	**5.80 [-0.53, 12.14]**	
Heterogeneity: Tau² = 107.69; Chi² = 10375.07, df = 10 (P < 0.00001); I² = 100%									
Test for overall effect: Z = 1.80 (P = 0.07)									
Test for subgroup differences: Chi² = 11.91, df = 1 (P = 0.0006), I² = 91.6%									

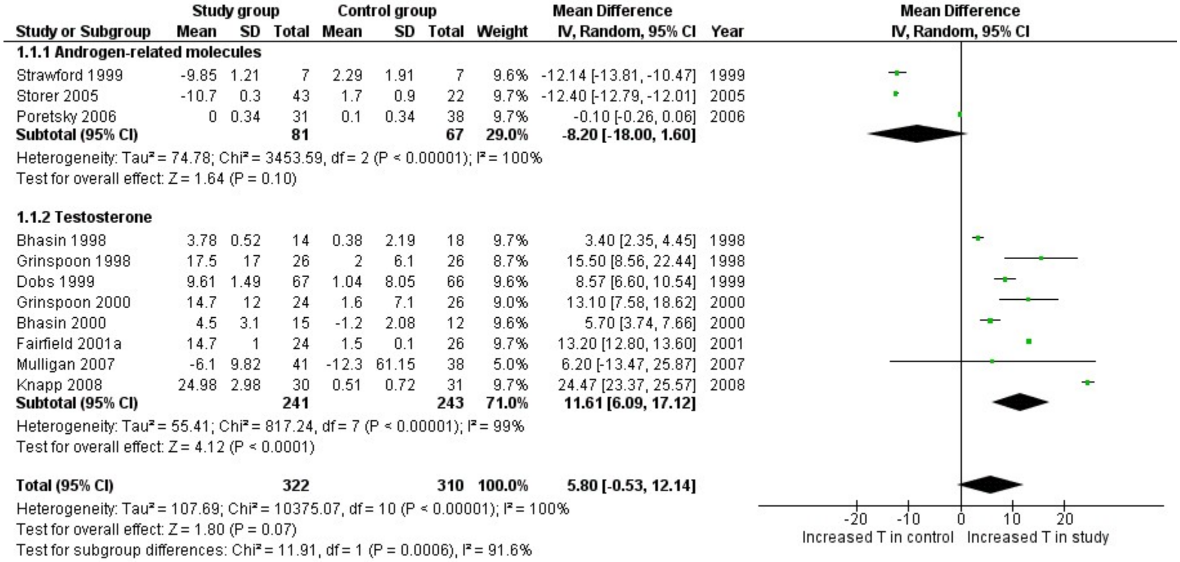

Panel A

Study or Subgroup	Study group Mean	SD	Total	Control group Mean	SD	Total	Weight	Mean Difference IV, Random, 95% CI	Year
**2.1.1 Non hypogonadal**									
Grinspoon 2000	14.7	12	24	1.6	7.1	26	12.5%	13.10 [7.58, 18.62]	2000
Fairfield 2001a	14.7	1	24	1.5	0.1	26	14.3%	13.20 [12.80, 13.60]	2001
Mulligan 2007	-6.1	9.82	41	-12.3	61.15	38	5.1%	6.20 [-13.47, 25.87]	2007
**Subtotal (95% CI)**			**89**			**90**	**31.8%**	**13.20 [12.80, 13.60]**	
Heterogeneity: Tau² = 0.00; Chi² = 0.49, df = 2 (P = 0.78); I² = 0%									
Test for overall effect: Z = 64.54 (P < 0.00001)									
**2.1.2 Hypogonadal**									
Bhasin 1998	3.78	0.52	14	0.38	2.19	18	14.2%	3.40 [2.35, 4.45]	1998
Grinspoon 1998	17.5	17	26	2	6.1	26	11.7%	15.50 [8.56, 22.44]	1998
Dobs 1999	9.61	1.49	67	1.04	8.05	66	14.0%	8.57 [6.60, 10.54]	1999
Bhasin 2000	4.5	3.1	15	-1.2	2.08	12	14.0%	5.70 [3.74, 7.66]	2000
Knapp 2008	24.98	2.98	30	0.51	0.72	31	14.2%	24.47 [23.37, 25.57]	2008
**Subtotal (95% CI)**			**152**			**153**	**68.2%**	**11.47 [1.19, 21.75]**	
Heterogeneity: Tau² = 134.70; Chi² = 808.24, df = 4 (P < 0.00001); I² = 100%									
Test for overall effect: Z = 2.19 (P = 0.03)									
**Total (95% CI)**			**241**			**243**	**100.0%**	**11.61 [6.09, 17.12]**	
Heterogeneity: Tau² = 55.41; Chi² = 817.24, df = 7 (P < 0.00001); I² = 99%									
Test for overall effect: Z = 4.12 (P < 0.0001)									
Test for subgroup differences: Chi² = 0.11, df = 1 (P = 0.74), I² = 0%									

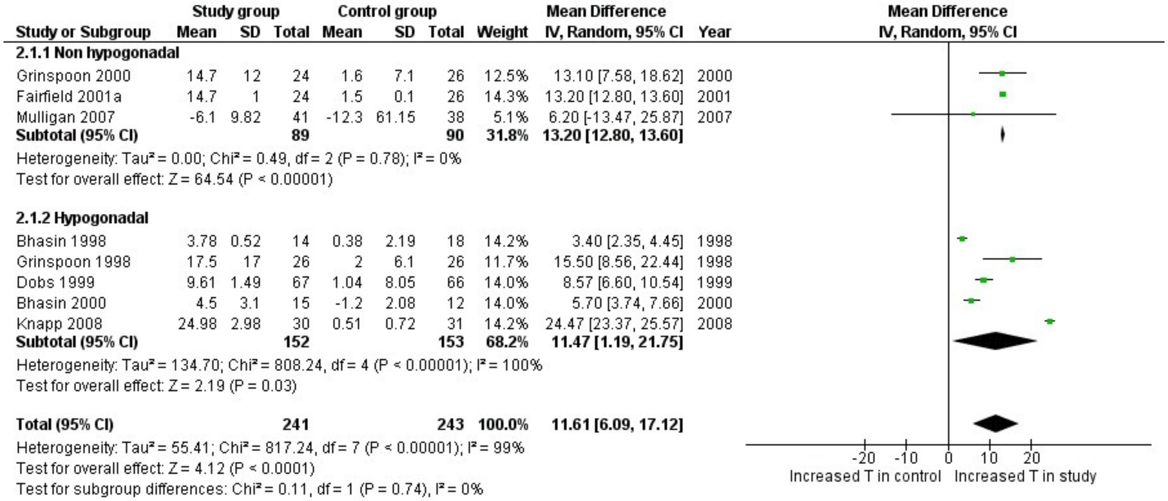

Panel B

Study or Subgroup	Study group Mean	SD	Total	Control group Mean	SD	Total	Weight	Mean Difference IV, Random, 95% CI	Year
**3.1.1 Wasting**									
Grinspoon 1998	17.5	17	26	2	6.1	26	11.7%	15.50 [8.56, 22.44]	1998
Dobs 1999	9.61	1.49	67	1.04	8.05	66	14.0%	8.57 [6.60, 10.54]	1999
Grinspoon 2000	14.7	12	24	1.6	7.1	26	12.5%	13.10 [7.58, 18.62]	2000
Bhasin 2000	4.5	3.1	15	-1.2	2.08	12	14.0%	5.70 [3.74, 7.66]	2000
Fairfield 2001a	14.7	1	24	1.5	0.1	26	14.3%	13.20 [12.80, 13.60]	2001
Mulligan 2007	-6.1	9.82	41	-12.3	61.15	38	5.1%	6.20 [-13.47, 25.87]	2007
Knapp 2008	24.98	2.98	30	0.51	0.72	31	14.2%	24.47 [23.37, 25.57]	2008
**Subtotal (95% CI)**			**227**			**225**	**85.8%**	**12.99 [7.37, 18.62]**	
Heterogeneity: Tau² = 48.89; Chi² = 464.46, df = 6 (P < 0.00001); I² = 99%									
Test for overall effect: Z = 4.53 (P < 0.00001)									
**3.1.2 Not Wasting**									
Bhasin 1998	3.78	0.52	14	0.38	2.19	18	14.2%	3.40 [2.35, 4.45]	1998
**Subtotal (95% CI)**			**14**			**18**	**14.2%**	**3.40 [2.35, 4.45]**	
Heterogeneity: Not applicable									
Test for overall effect: Z = 6.36 (P < 0.00001)									
**Total (95% CI)**			**241**			**243**	**100.0%**	**11.61 [6.09, 17.12]**	
Heterogeneity: Tau² = 55.41; Chi² = 817.24, df = 7 (P < 0.00001); I² = 99%									
Test for overall effect: Z = 4.12 (P < 0.0001)									
Test for subgroup differences: Chi² = 10.80, df = 1 (P = 0.001), I² = 90.7%									

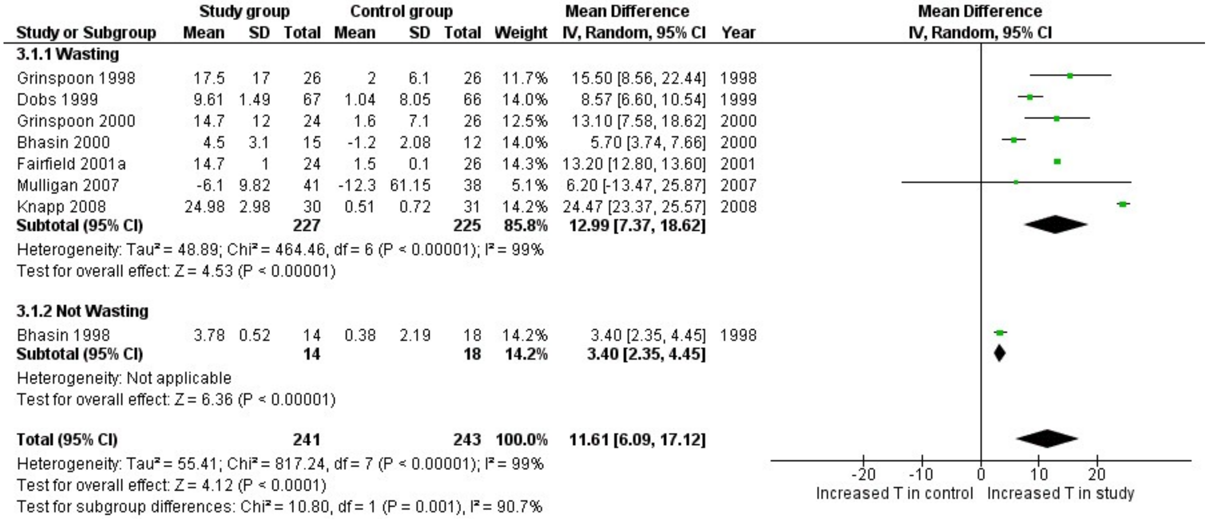

Panel C

Supplementary Figure S3.(Panel A Panel B Panel C)

Study or Subgroup	Study group Mean	SD	Total	Control group Mean	SD	Total	Weight	Mean Difference IV, Random, 95% CI	Year
**1.10.1 Androgen-related molecules**									
Storer 2005	-91.2	24.4	43	14.2	14.3	22	13.7%	-105.40 [-114.83, -95.97]	2005
Poretsky 2006	41.6	0.03	31	-6.94	0.1	38	14.1%	48.54 [48.51, 48.57]	2006
**Subtotal (95% CI)**			**74**			**60**	**27.8%**	**-28.35 [-179.21, 122.50]**	
Heterogeneity: Tau² = 11837.19; Chi² = 1024.05, df = 1 (P < 0.00001); I² = 100%									
Test for overall effect: Z = 0.37 (P = 0.71)									
**1.10.2 Testosterone**									
Bhasin 1998	7	1	14	2	2	18	14.1%	5.00 [3.94, 6.06]	1998
Grinspoon 1998	68	63	26	6	17	26	12.0%	62.00 [36.92, 87.08]	1998
Dobs 1999	3.8	4.1	67	3.5	4.3	66	14.1%	0.30 [-1.13, 1.73]	1999
Grinspoon 2000	66	53	24	0	23	26	12.3%	66.00 [43.03, 88.97]	2000
Bhasin 2000	148	107.28	15	1	83.83	12	5.8%	147.00 [74.91, 219.09]	2000
Knapp 2008	6.52	1.3	30	-7	3.1	31	14.1%	13.52 [12.33, 14.71]	2008
**Subtotal (95% CI)**			**176**			**179**	**72.2%**	**16.11 [8.64, 23.58]**	
Heterogeneity: Tau² = 51.24; Chi² = 272.42, df = 5 (P < 0.00001); I² = 98%									
Test for overall effect: Z = 4.22 (P < 0.0001)									
**Total (95% CI)**			**250**			**239**	**100.0%**	**19.02 [-3.54, 41.57]**	
Heterogeneity: Tau² = 941.32; Chi² = 15186.25, df = 7 (P < 0.00001); I² = 100%									
Test for overall effect: Z = 1.65 (P = 0.10)									
Test for subgroup differences: Chi² = 0.33, df = 1 (P = 0.56), I² = 0%									

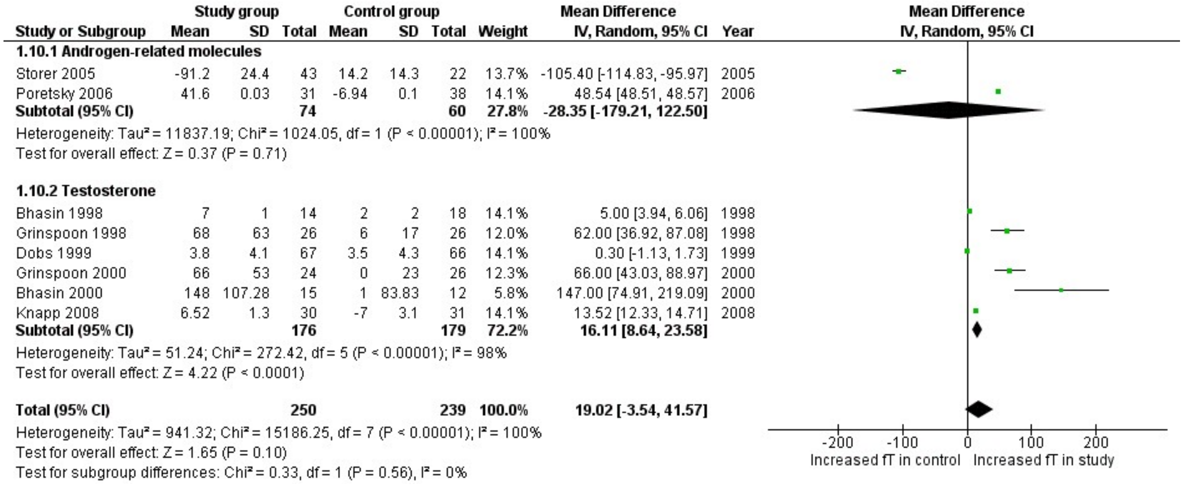

Supplementary Figure 4

Study or Subgroup	Study group Mean	SD	Total	Control group Mean	SD	Total	Weight	Mean Difference IV, Random, 95% CI	Year
**2.3.1 Non hypogonadal**									
Coodley 1997	-0.12	0.1	17	0.87	2	18	10.6%	-0.99 [-1.92, -0.06]	1997
Grinspoon 2000	2.6	2.5	24	0.4	3	26	8.2%	2.20 [0.67, 3.73]	2000
Grunfeld 2006	2.3	2.9	64	1.1	2.7	65	10.4%	1.20 [0.23, 2.17]	2006
Mulligan 2007	5.3	11.4	41	7.3	6.08	38	2.7%	-2.00 [-5.99, 1.99]	2007
Sardar 2010	1.55	2.35	42	0.5	1.55	20	10.4%	1.05 [0.07, 2.03]	2010
**Subtotal (95% CI)**			**188**			**167**	**42.3%**	**0.59 [-0.66, 1.85]**	
Heterogeneity: Tau² = 1.45; Chi² = 19.43, df = 4 (P = 0.0006); I² = 79%									
Test for overall effect: Z = 0.92 (P = 0.36)									
**2.3.2 Hypogonadal**									
Grinspoon 1998	1.6	5.8	26	-0.3	5.5	26	3.9%	1.90 [-1.17, 4.97]	1998
Bhasin 1998	0.6	0.5	14	0.6	0.7	18	12.2%	0.00 [-0.42, 0.42]	1998
Grinspoon 1999	2.9	0.51	26	-0.3	5	26	6.8%	3.20 [1.27, 5.13]	1999
Dobs 1999	0.8	2.87	67	1	2.85	66	10.4%	-0.20 [-1.17, 0.77]	1999
Bhasin 2000	2.9	0.8	15	0.7	0.7	12	11.8%	2.20 [1.63, 2.77]	2000
Knapp 2008	1.8	0.6	30	0.2	0.6	31	12.5%	1.60 [1.30, 1.90]	2008
**Subtotal (95% CI)**			**178**			**179**	**57.7%**	**1.27 [0.30, 2.24]**	
Heterogeneity: Tau² = 1.11; Chi² = 62.97, df = 5 (P < 0.00001); I² = 92%									
Test for overall effect: Z = 2.56 (P = 0.01)									
**Total (95% CI)**			**366**			**346**	**100.0%**	**0.99 [0.25, 1.72]**	
Heterogeneity: Tau² = 1.10; Chi² = 87.55, df = 10 (P < 0.00001); I² = 89%									
Test for overall effect: Z = 2.64 (P = 0.008)									
Test for subgroup differences: Chi² = 0.70, df = 1 (P = 0.40), I² = 0%									

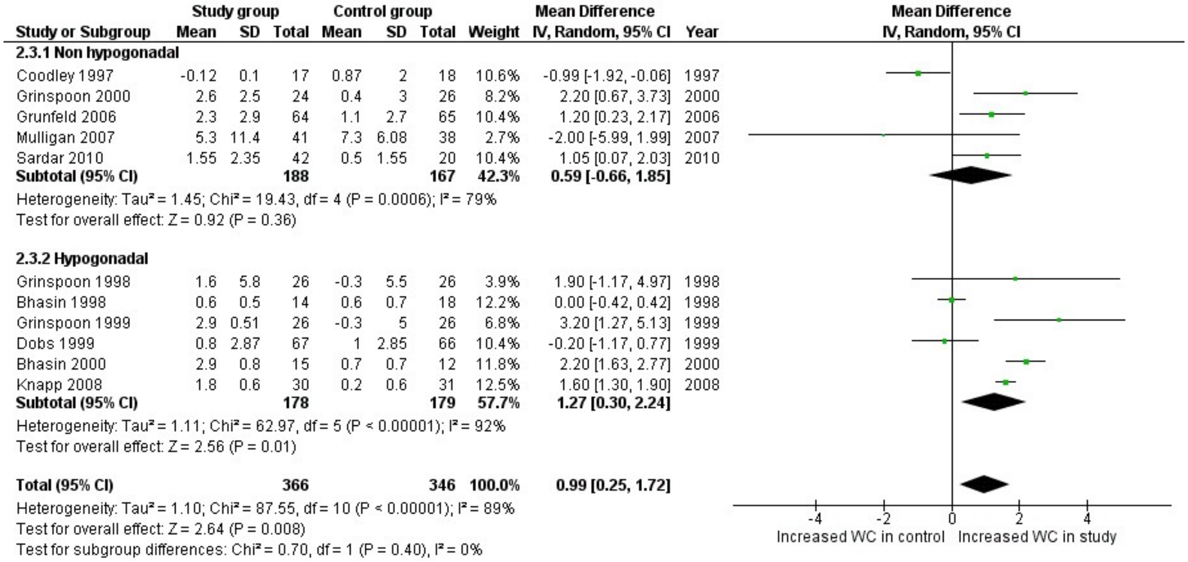

Panel A

Study or Subgroup	Study group Mean	SD	Total	Control group Mean	SD	Total	Weight	Mean Difference IV, Random, 95% CI	Year
**3.3.1 Wasting**									
Coodley 1997	-0.12	0.1	17	0.87	2	18	10.6%	-0.99 [-1.92, -0.06]	1997
Grinspoon 1998	1.6	5.8	26	-0.3	5.5	26	3.9%	1.90 [-1.17, 4.97]	1998
Dobs 1999	0.8	2.87	67	1	2.85	66	10.4%	-0.20 [-1.17, 0.77]	1999
Grinspoon 1999	2.9	0.51	26	-0.3	5	26	6.8%	3.20 [1.27, 5.13]	1999
Bhasin 2000	2.9	0.8	15	0.7	0.7	12	11.8%	2.20 [1.63, 2.77]	2000
Grinspoon 2000	2.6	2.5	24	0.4	3	26	8.2%	2.20 [0.67, 3.73]	2000
Grunfeld 2006	2.3	2.9	64	1.1	2.7	65	10.4%	1.20 [0.23, 2.17]	2006
Mulligan 2007	5.3	11.4	41	7.3	6.08	38	2.7%	-2.00 [-5.99, 1.99]	2007
Knapp 2008	1.8	0.6	30	0.2	0.6	31	12.5%	1.60 [1.30, 1.90]	2008
Sardar 2010	1.55	2.35	42	0.5	1.55	20	10.4%	1.05 [0.07, 2.03]	2010
**Subtotal (95% CI)**			**352**			**328**	**87.8%**	**1.13 [0.39, 1.86]**	
Heterogeneity: Tau² = 0.94; Chi² = 53.32, df = 9 (P < 0.00001); I² = 83%									
Test for overall effect: Z = 2.99 (P = 0.003)									
**3.3.2 Non Wasting**									
Bhasin 1998	0.6	0.5	14	0.6	0.7	18	12.2%	0.00 [-0.42, 0.42]	1998
**Subtotal (95% CI)**			**14**			**18**	**12.2%**	**0.00 [-0.42, 0.42]**	
Heterogeneity: Not applicable									
Test for overall effect: Z = 0.00 (P = 1.00)									
**Total (95% CI)**			**366**			**346**	**100.0%**	**0.99 [0.25, 1.72]**	
Heterogeneity: Tau² = 1.10; Chi² = 87.55, df = 10 (P < 0.00001); I² = 89%									
Test for overall effect: Z = 2.64 (P = 0.008)									
Test for subgroup differences: Chi² = 6.79, df = 1 (P = 0.009), I² = 85.3%									

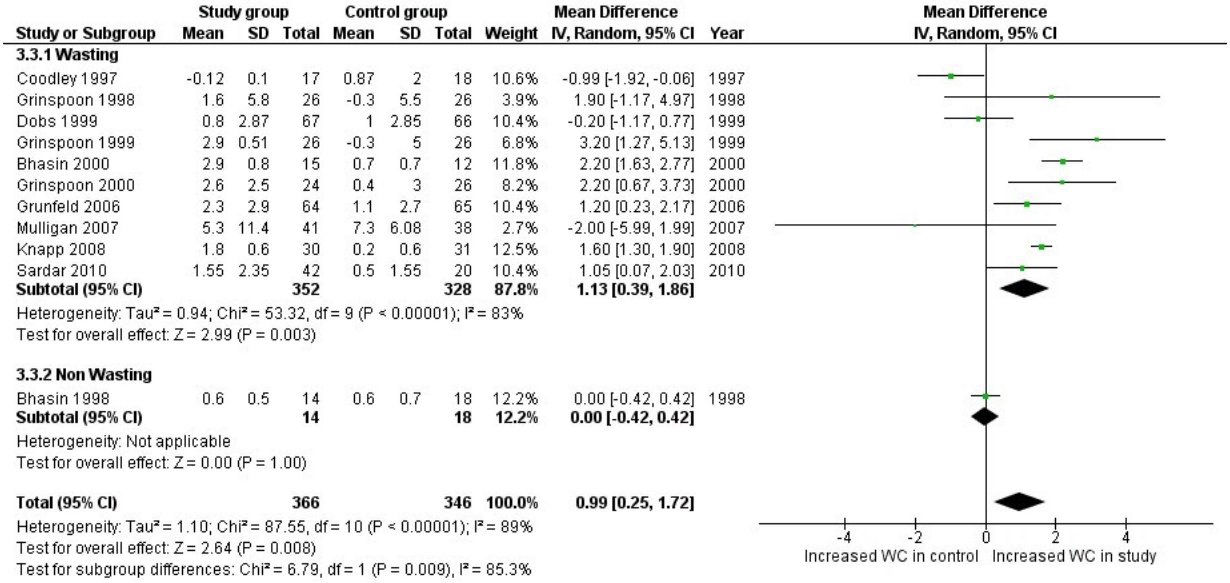

Panel B

Supplementary Figure S5.(Panel A Panel B)

Study or Subgroup	Study group Mean	Study group SD	Study group Total	Control group Mean	Control group SD	Control group Total	Weight	Mean Difference IV, Random, 95% CI	Year
**2.6.1 Non hypogonadal**									
Grinspoon 2000	4.4	2.2	24	1	2.7	26	18.4%	3.40 [2.04, 4.76]	2000
Mulligan 2007	3.3	3.8	41	3.3	2.43	38	18.1%	0.00 [-1.40, 1.40]	2007
**Subtotal (95% CI)**			**65**			**64**	**36.5%**	**1.70 [-1.63, 5.04]**	
Heterogeneity: Tau² = 5.29; Chi² = 11.68, df = 1 (P = 0.0006); I² = 91%									
Test for overall effect: Z = 1.00 (P = 0.32)									
**2.6.2 Hypogonadal**									
Bhasin 1998	2.15	0.3	14	0.63	0.16	18	25.1%	1.52 [1.35, 1.69]	1998
Grinspoon 1998	1.9	3.4	26	0	2.9	26	15.8%	1.90 [0.18, 3.62]	1998
Dobs 1999	0.3	2.46	67	0.6	2.03	66	22.6%	-0.30 [-1.07, 0.47]	1999
**Subtotal (95% CI)**			**107**			**110**	**63.5%**	**0.97 [-0.43, 2.36]**	
Heterogeneity: Tau² = 1.27; Chi² = 20.91, df = 2 (P < 0.0001); I² = 90%									
Test for overall effect: Z = 1.36 (P = 0.17)									
**Total (95% CI)**			**172**			**174**	**100.0%**	**1.24 [0.12, 2.36]**	
Heterogeneity: Tau² = 1.29; Chi² = 32.96, df = 4 (P < 0.00001); I² = 88%									
Test for overall effect: Z = 2.17 (P = 0.03)									
Test for subgroup differences: Chi² = 0.16, df = 1 (P = 0.69), I² = 0%									

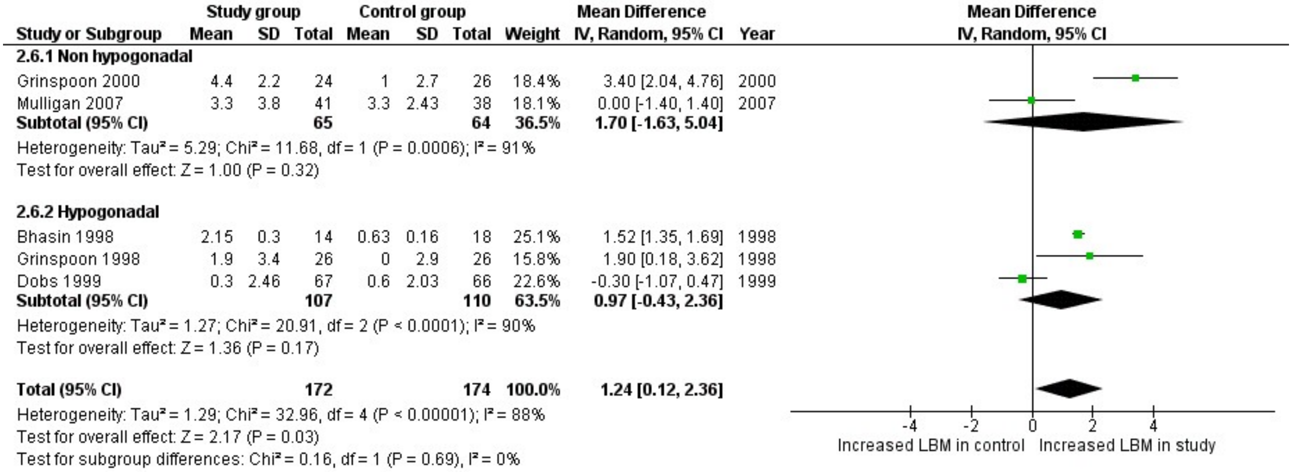

Panel A

Study or Subgroup	Study group Mean	Study group SD	Study group Total	Control group Mean	Control group SD	Control group Total	Weight	Mean Difference IV, Random, 95% CI	Year
**3.6.1 Wasting**									
Grinspoon 1998	1.9	3.4	26	0	2.9	26	15.8%	1.90 [0.18, 3.62]	1998
Dobs 1999	0.3	2.46	67	0.6	2.03	66	22.6%	-0.30 [-1.07, 0.47]	1999
Grinspoon 2000	4.4	2.2	24	1	2.7	26	18.4%	3.40 [2.04, 4.76]	2000
Mulligan 2007	3.3	3.8	41	3.3	2.43	38	18.1%	0.00 [-1.40, 1.40]	2007
**Subtotal (95% CI)**			**158**			**156**	**74.9%**	**1.20 [-0.59, 2.99]**	
Heterogeneity: Tau² = 2.88; Chi² = 24.46, df = 3 (P < 0.0001); I² = 88%									
Test for overall effect: Z = 1.31 (P = 0.19)									
**3.6.2 Non Wasting**									
Bhasin 1998	2.15	0.3	14	0.63	0.16	18	25.1%	1.52 [1.35, 1.69]	1998
**Subtotal (95% CI)**			**14**			**18**	**25.1%**	**1.52 [1.35, 1.69]**	
Heterogeneity: Not applicable									
Test for overall effect: Z = 17.15 (P < 0.00001)									
**Total (95% CI)**			**172**			**174**	**100.0%**	**1.24 [0.12, 2.36]**	
Heterogeneity: Tau² = 1.29; Chi² = 32.96, df = 4 (P < 0.00001); I² = 88%									
Test for overall effect: Z = 2.17 (P = 0.03)									
Test for subgroup differences: Chi² = 0.12, df = 1 (P = 0.73), I² = 0%									

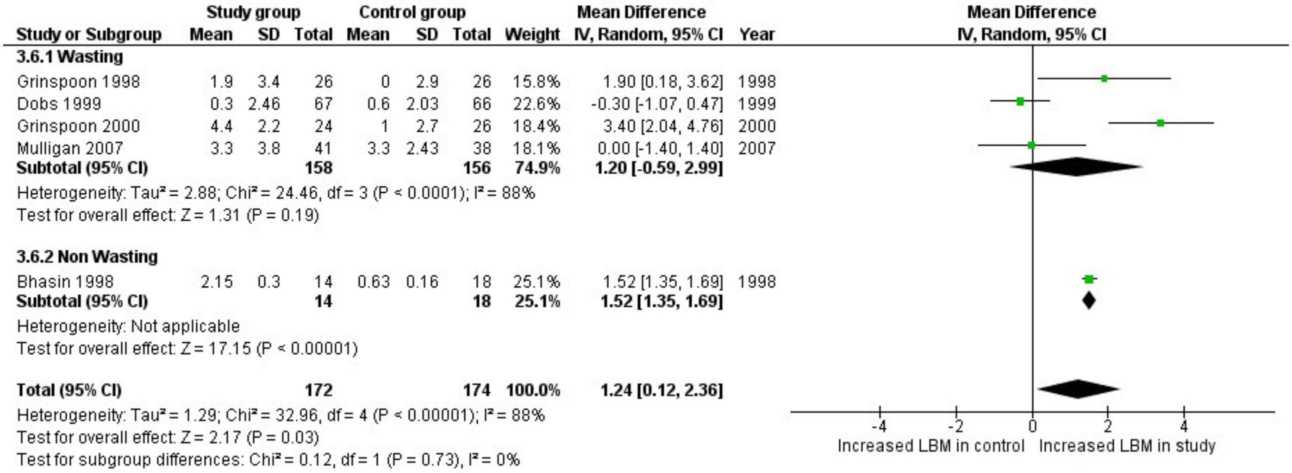

Panel B

Supplementary Figure S6.(Panel A Panel B)

Study or Subgroup	Study group Mean	Study group SD	Study group Total	Control group Mean	Control group SD	Control group Total	Weight	Mean Difference IV, Random, 95% CI	Year
**1.8.1 Nandrolone**									
Strawford 1999	1.7	2.8	11	1.6	1.9	11	4.3%	0.10 [-1.90, 2.10]	1999
Storer 2005	0.1	0.2	43	0.3	0.4	22	32.6%	-0.20 [-0.38, -0.02]	2005
Grunfeld 2006	0.6	1.8	64	0.3	1.6	65	21.4%	0.30 [-0.29, 0.89]	2006
**Subtotal (95% CI)**			**118**			**98**	**58.3%**	**-0.08 [-0.40, 0.24]**	
Heterogeneity: Tau² = 0.03; Chi² = 2.61, df = 2 (P = 0.27); I² = 23%									
Test for overall effect: Z = 0.48 (P = 0.63)									
**1.8.2 Testosterone**									
Grinspoon 1998	0.9	2.7	26	0.3	3.6	26	5.5%	0.60 [-1.13, 2.33]	1998
Grinspoon 1999	0.9	3.57	26	0.3	3.5	25	4.5%	0.60 [-1.34, 2.54]	1999
Bhasin 2000	0.2	2.32	15	-0.1	2.42	12	5.1%	0.30 [-1.50, 2.10]	2000
Grinspoon 2000	-2.4	2.3	24	-0.8	2.6	26	8.1%	-1.60 [-2.96, -0.24]	2000
Mulligan 2007	3	3.8	41	3.8	3.95	38	5.6%	-0.80 [-2.51, 0.91]	2007
Sardar 2010	1.41	2.96	42	0.43	0.92	20	12.8%	0.98 [-0.00, 1.96]	2010
**Subtotal (95% CI)**			**174**			**147**	**41.7%**	**0.02 [-0.90, 0.94]**	
Heterogeneity: Tau² = 0.68; Chi² = 10.78, df = 5 (P = 0.06); I² = 54%									
Test for overall effect: Z = 0.04 (P = 0.97)									
**Total (95% CI)**			**292**			**245**	**100.0%**	**0.03 [-0.41, 0.47]**	
Heterogeneity: Tau² = 0.15; Chi² = 14.14, df = 8 (P = 0.08); I² = 43%									
Test for overall effect: Z = 0.13 (P = 0.90)									
Test for subgroup differences: Chi² = 0.04, df = 1 (P = 0.84), I² = 0%									

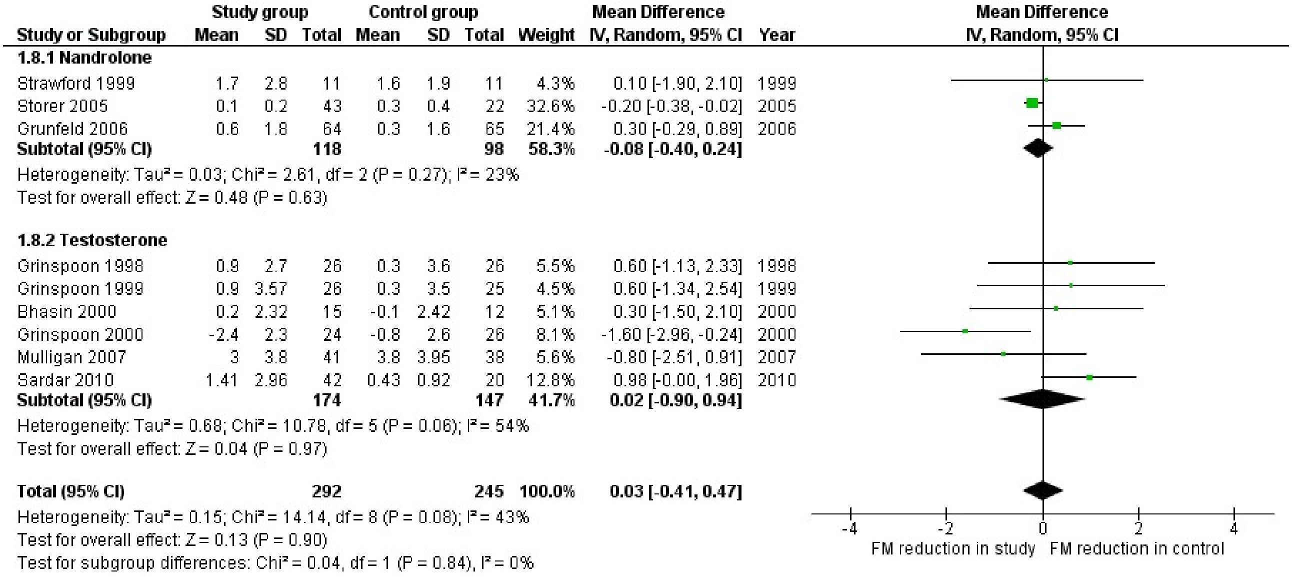

Panel A

Study or Subgroup	Study group Mean	Study group SD	Study group Total	Control group Mean	Control group SD	Control group Total	Weight	Mean Difference IV, Random, 95% CI	Year
**2.8.1 Non hypogonadal**									
Grinspoon 2000	-2.4	2.3	24	-0.8	2.6	26	18.8%	-1.60 [-2.96, -0.24]	2000
Mulligan 2007	3	3.8	41	3.8	3.95	38	15.2%	-0.80 [-2.51, 0.91]	2007
**Subtotal (95% CI)**			**65**			**64**	**34.0%**	**-1.29 [-2.35, -0.23]**	
Heterogeneity: Tau² = 0.00; Chi² = 0.51, df = 1 (P = 0.47); I² = 0%									
Test for overall effect: Z = 2.38 (P = 0.02)									
**2.8.2 Hypogonadal**									
Grinspoon 1998	0.9	2.7	26	0.3	3.6	26	15.0%	0.60 [-1.13, 2.33]	1998
Grinspoon 1999	0.9	3.57	26	0.3	3.5	25	13.2%	0.60 [-1.34, 2.54]	1999
Bhasin 2000	0.2	2.32	15	-0.1	2.42	12	14.3%	0.30 [-1.50, 2.10]	2000
Sardar 2010	1.41	2.96	42	0.43	0.92	20	23.5%	0.98 [-0.00, 1.96]	2010
**Subtotal (95% CI)**			**109**			**83**	**66.0%**	**0.76 [0.04, 1.47]**	
Heterogeneity: Tau² = 0.00; Chi² = 0.50, df = 3 (P = 0.92); I² = 0%									
Test for overall effect: Z = 2.06 (P = 0.04)									
**Total (95% CI)**			**174**			**147**	**100.0%**	**0.02 [-0.90, 0.94]**	
Heterogeneity: Tau² = 0.68; Chi² = 10.78, df = 5 (P = 0.06); I² = 54%									
[illegible]									

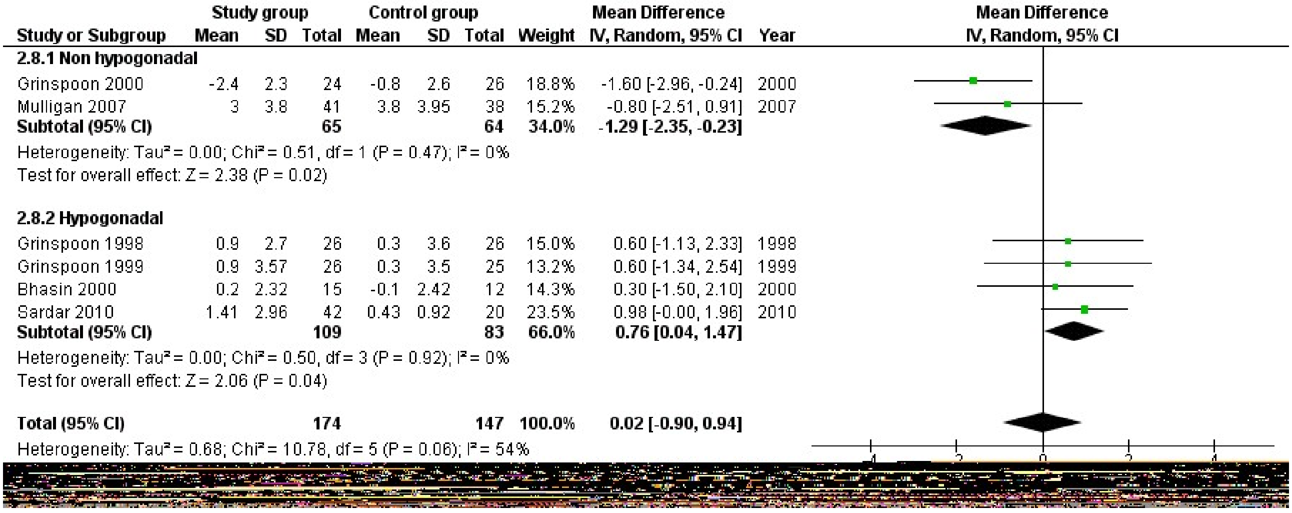

Panel B

Supplementary Figure S7.(Panel A Panel B)
